# Health services performance for TB treatment in Brazil: a cross-sectional study

**DOI:** 10.1186/1472-6963-11-241

**Published:** 2011-09-28

**Authors:** Tereza CS Villa, Antônio Ruffino-Netto, Lucia M Scatena, Rubia LP Andrade, Maria EF Brunello, Jordana A Nogueira, Pedro F Palha, Lenilde D Sá, Marluce MA Assis, Silvia HF Vendramini, Aline A Monroe, Ricardo A Arcêncio, Tiemi Arakawa

**Affiliations:** 1 Ribeirao Preto College of Nursing, University of São Paulo, Avenida Bandeirantes, 3900 - Campus Universitário - Ribeirão Preto, SP, CEP: 14040-902, Brazil; 2 Departamento de Medicina Social, Ribeirao Preto School of Medicine, University of São Paulo, Ribeirão Preto, SP, Brazil; 3 Departamento de Medicina Social, Triangulo Mineiro Federal University, Uberaba, MG, Brazil; 4 Ribeirao Preto College of Nursing, University of São Paulo, Ribeirão Preto, SP, Brazil; 5 Departamento de Enfermagem Clínica, Paraíba Federal University, João Pessoa, PB, Brazil; 6 Departamento de Enfermagem em Saúde Pública e Psiquiátrica, Paraíba Federal University, João Pessoa, PB, Brazil; 7 State University of Feira de Santana, Feira de Santana, BA, Brazil; 8 Departamento de saúde Coletiva e Orientação Profissional, São José do Rio Preto School of Medicine, São José do Rio Preto, SP, Brazil

**Keywords:** Primary Healthcare, Tuberculosis, prevention & control, Health Care, Health Services Accessibility, Health Services Evaluation

## Abstract

**Background:**

Researches to evaluate Primary Health Care performance in TB control in Brazil show that different cities aggregate local specificities in the dynamics of coping with the disease. This study aims to evaluate health services' performance in TB treatment in cities across different Brazilian regions.

**Methods:**

This cross-sectional study was conducted in five cities that are considered priorities for TB control in Brazil: Itaboraí (ITA), Ribeirão Preto (RP) and São José do Rio Preto (SJRP) in the Southeast; Campina Grande (CG) and Feira de Santana (FS) in the Northeast. Data were collected through interviews with 514 TB patients under treatment in 2007, using the *Primary Care Assessment Tool *adapted for TB care in Brazil. Indicators were constructed based on the mean response scores (Likert scale) and compared among the study sites.

**Results:**

"Access to treatment" was evaluated as satisfactory in the Southeast and regular in the Northeast, which displayed poor results on 'home visits' and 'distance between treatment site and patient's house'. "Bond" was assessed as satisfactory in all cities, with a slightly better performance in RP and SJRP. "Range of services" was rated as regular, with better performance of southeastern cities. 'Health education', 'DOT' and 'food vouchers' were less offered in the Northeast. "Coordination" was evaluated as satisfactory in all cities. "Family focus" was evaluated as satisfactory in RP and SJRP, and regular in the others. 'Professional asking patient's family about other health problems' was evaluated as unsatisfactory, except in RP.

**Conclusions:**

Two types of obstacles are faced for health service performance in TB treatment in the cities under analysis, mainly in the Northeast. The first is structural and derives from difficulties to access health services and actions. The second is organizational and derives from the way health technologies and services are distributed and integrated. Incentives to improve care organization and management practices, aimed at the integration of primary, secondary and tertiary services, can contribute towards a better performance of health services in TB treatment.

## Background

Even in this millennium, tuberculosis (TB) remains the leading killer infectious disease in the world, with 1.7 million deaths in 2009. One third of the world population is infected by Mycobacterium tuberculosis and a great proportion of the population may develop and transmit the disease to the community [1].

Brazil ranks 19th among the 22 countries with the highest incidence levels of tuberculosis (TB) smear-positive cases [1]. The TB incidence rate was 46 cases/100, 000 inhabitants in 2009, considered one of the highest on the American continent. Although the prevalence and mortality the disease provokes have dropped and cure percentages have increased, from 69% in 2002 to 71% in 2009, this rate still remains far below the recommended 85% [1].

Brazil has considerably invested in health system reform, including the development of new primary health care (PHC) organization and delivery models [2]. The country has recommended TB control as a responsibility of this care level [3,4]. To achieve service quality, PHC attributes (access, range of services, coordination, bond and family focus) need to be accomplished, so that a good organization of this level contributes to improve care, with a view to positive impacts on population health and health system efficiency [5].

Despite this recommendation, in some contexts, TB treatment remains centralized in TB reference centers (TRC), so that it has not yet resulted in a uniform performance of health care services in TB control, varying between and within regions.

Disease control cannot be achieved through medical advances alone, such as new diagnostic tests and new drugs. It is important to consider the scenarios and complexity of health care services' context, where technologies are actually incorporated and offered to the community. Currently, research is also lacking a detailed analysis of the interaction between available technologies for TB control and the diversity of local health system contexts, considering the resources, political project and the willingness of local health managers and health care workers [6,7].

Research on the evaluation of PHC performance in TB control in Brazil show that the different care models present in the cities aggregate local (political/organizational/human) specificities, entailing heterogeneity in the dynamics of coping with the disease [8-10].

Considering the diversity of health local systems, regional disparities and inequalities in access to health services that characterize the Brazilian reality, it is appropriate to investigate how health care services carry out TB treatment actions. Thus, this study proposed to evaluate the performance of health services in TB treatment in cities from different Brazilian regions.

## Methods

### Research Design and Setting

A cross-sectional study was conducted in five cities from two Brazilian regions (3 in the Southeast and 2 in the Northeast). These cities were intentionally selected due to their epidemiological and operational TB status, as the National TB Control Program considers them priority cities for disease control in their regions, including implementation of the Directly Observed Therapy Short-Course (DOTS) strategy for at least five years.

Ribeirão Preto (RP) and São José do Rio Preto (SJRP), in São Paulo State, and Itaboraí (ITA), in Rio de Janeiro State, are the Southeastern study settings. In 2007, TB incidence in those cities was: 29 cases/100, 000 inhabitants (RP); 32.3 cases/100, 000 inhabitants (SJRP) and 56.1 cases/100, 000 inhabitants (ITA). The Northeastern study sites were Campina Grande (CG), in Paraíba State, and Feira de Santana (FS), in Bahia State, which reached a TB incidence rate of 28.6 cases/100, 000 inhabitants and 28.1 cases/100, 000 inhabitants, respectively [11]. In the same year, TB incidence was 40.6/100, 000 inhabitants in the Southeast and 38.8/100, 000 inhabitants in the Northeast.

Regarding PHC organization, in 2007, FHS coverage corresponded to 23% in RP, 12% in SJRP and 68.7% in ITA. In the Northeast, CG obtained 71% of FHS coverage and FS 60%. The implementation of the FHS has been taking shape in a heterogeneous way in Brazil, with a team composed by a general practitioner, a nurse, a nursing auxiliaries and 5 to 10 Community Health Workers (CHW), who see to a clientele in a given geographical area.

Historically, a great expansion of the FHS has been observed in areas with poor health resources, aiming to enhance access to health services [12]. This fact has led to a higher FHS coverage rate in the Northeast. In the Southeast, traditional models are still prevalent, structured in Basic Health Units (BHU), in which the teams comprise specialist medical practitioners, nurses and nursing auxiliaries.

As for TB care organization, two types of care models are identified at the study sites: TB care centralized at TB Reference Centers - TRC (RP and FS) or partly decentralized at PHC services (ITA, SJRP and CG).

At TRC, specialized teams conduct TB care with a centralized organization, greater availability of health equipments and provision of diagnostic examinations.

PHC refers to the first level of care provided in the health system and comprises the Family Health Strategy Units (FHS) and Basic Health Units (BHU). Generalist teams conduct TB care in PHC, with lower availability of technologies.

### Participants and Sampling

The study population was composed of TB patients under treatment in 2007. Patients younger than 18 years (due to ethical considerations) and prison population (due to differences in treatment conduction) were excluded.

The sample size required for ANOVA was 98 patients for each city, calculated using *Statistica *software (using the commands Several means, ANOVA, 1-Way) and considering the parameters: number of cities = 5; probability of type I error = 0.05; probability of type II error = 0.10, variation due to error = 0.2. Data collection was conducted over three months (July to September 2007) until the minimum sample size was reached.

From July to September 2007, 618 patients were under treatment for TB, 514 of whom met the inclusion criteria and agreed to participate in the survey (100 from RP, 108 from SJRP, 100 from ITA, 106 from CG and 100 from FS).

### Data Collection Tool

To address the issues raised, the theoretical framework of PHC dimensions [13] and the Primary Care Assessment Tool (PCAT) [13] were used, validated in Brazil [14] to evaluate PHC organization and performance and adapted for TB care evaluation [15]. A pilot study was carried out previously.

The instrument to evaluate health service performance in TB control contains questions regarding socio-demographic, clinical and epidemiological aspects, site and type of treatment and specific questions for each organizational PHC component of TB control, divided into five sections:

I - "access to treatment": which involves the location of the health unit near the population it attends, the times and days it is open to treat patients, the degree of tolerance for unscheduled consultations and how the population perceives the convenience of these access aspects [13].

II - "bond" (longitudinality): which implies the existence of a regular source of care and its use over time and requires the establishment of interpersonal bonds that reflect the mutual cooperation between community people and health professionals [13].

III - "range of services" (comprehensiveness): this represents arrangements for the patient to receive any kind of healthcare services required [13].

IV - "coordination": this implies some form of continuity, either care by the same professional, through medical records or both, as well as the recognition of past and new problems. This also includes referral and follow-up care at other specialized services [13].

V - "family focus": which considers the context and family dynamics in care [13].

Interviews with TB patients were conducted to complete the instrument. Patients were interviewed as soon as they went to the health services to perform the medical consultation or DOT, or during home visits. Interviewees should answer each question according to a variety of response scales, such as dichotomous, multiple choice with single answers and a five-point Likert scale, with higher scores for positive responses.

### Analysis

For data analysis, frequency distribution was used to describe the patients' socio-demographical and clinical profile. To analyze the study dimensions, indicators were constructed based on the variables (questions) created for each. The indicators corresponded to the mean value obtained by adding up all responses of all interviewees to each question, divided by the total number of interviewees. Subsequently, an overall indicator was specified for a general analysis of the dimensions, using the mean of responses to all questions pertaining to each study dimension. To test the hypothesis about the existence or lack of similarity between the overall indicators for different cities and regions, analysis of variance (ANOVA), Tukey's test and Student's t-test were performed. The criterion of homoscedasticity was verified using Bartlett's test. The performance of the indicators and dimensions was classified according to the values obtained. Values between 1 and 2 were considered unsatisfactory performance, close to 3, regular and between 4 and 5, satisfactory.

### Ethical aspects

Approval for the project was obtained from the Institutional Review Board at the Ribeirão Preto College of Nursing, University of São Paulo (EERP/USP).

## Results

The results showed a higher percentage of male patients (65.6%), patients with less than 8 years of education (51.1%) and those with: home ownership (67.9%), electricity (98.8%), public water supply at home (86.7%) and telephone (70.2%). The percentage was lower for patients with a private car (26.9%).

In total, 103 (20.0%) patients received treatment at PHC services and 411 (80.0%) at TRC. Of 514 patients interviewed, 271 (52.3%) carried out DOT, with 73.8% at PHC and 47.5% at TRC.

The results regarding the types of services that perform TB treatment and DOT coverage are presented per city:

• RP carried out treatment at TRC, 100 (100.0%); with DOT coverage, 81 (81.0%);

• SJRP performed treatment at TRC, 81 (75.0%), and PHC, 27 (25.0%); with 90 (83.3%) of DOT coverage;

• ITA performed treatment at PHC, 44 (44.0%), and TRC, 56 (56.0%); with 81 (81.0%) of DOT coverage;

• CG performed treatment at PHC, 32 (30.2%), and TRC, 74 (69.8%); with 17 (16.0%) of DOT coverage;

• FS performed treatment at TRC, 100 (100.0%), and did not carry out DOT.

The overall indicators calculated for the dimensions "access to treatment", "bond", "range of services", "coordination" and "family focus" are presented in tables 1, 2, 3, 4 and 5, respectively. Southeastern cities displayed better performance in all dimensions.

**Table 1:** Distribution of means and standard deviations of *treatment access *indicators, Brazilian municipalities, 2007.

Indicators of Treatment access	Southeastern cities			Northeastern cities	
	RP	SJRP	ITA	FS	CG
	Mean ± SD	Mean ± SD	Mean ± SD	Mean ± SD	Mean ± SD
Can you obtain a medical consultation within 24 hours?	4.59 ± 1.27	4.47 ± 1.21	4.21 ± 1.42	4.34 ± 1.23	4.01 ± 1.33
Do you lose a day of work due to the consultation?	3.51 ± 1.82	3.22 ± 1.79	3.84 ± 1.72	2.89 ± 1.94	2.75 ± 1.71
Do you use any type of motorized transport to go to the consultation?	1.93 ± 1.65	1.66 ± 1.42	2.21 ± 1.78	1.71 ± 1.49	2.33 ± 1.83
Do you pay for transport to go to the consultation?	3.33 ± 1.92	2.73 ± 1.86	2.40 ± 1.86	2.44 ± 1.82	2.62 ± 1.81
During your treatment, has TB medication been lacking?	4.82 ± 0.63	4.93 ± 0.30	4.84 ± 0.71	4.93 ± 0.43	4.89 ± 0.40
Have you waited more than 60 minutes to be seen for the consultation?	4.14 ± 1.30	4.45 ± 0.85	4.08 ± 1.40	3.98 ± 1.44	3.08 ± 1.32
Does the health professional often visit you at your home?	3.85 ± 1.43	2.94 ± 1.60	2.52 ± 1.81	1.80 ± 1.28	1.59 ± 1.23
Do you do the treatment at the HU closest to your house?	2.77 ± 1.99	2.90 ± 1.70	4.16 ± 1.61	1.44 ± 1.20	2.13 ± 1.72

**Total Indicator of Treatment access**	3.64 ± 1.77^a^	3.41 ± 1.76^a^	3.53 ± 1.83^a^	2.94 ± 1.88^b^	2.93 ± 1.79^b^

**Treatment access by region**	3.53 ± 1.79*			2.93 ± 1.83*	

RP - Ribeirão Preto; FS - Feira de Santana; CG - Campina Grande; SJRP - São José do Rio Preto; ITA - Itaboraí.

Note: No statistical differences were found among averages followed by the same letter in columns according to the Tukey test, with significance set at 5%.

*Student t-test - p < 0.05

**Table 2:** Distribution of means and standard deviations of *bond *indicators, Brazilian municipalities, 2007.

Indicators of Bond	Southeastern cities			Northeastern cities	
	RP	SJRP	ITA	FS	CG
	Mean ± SD	Mean ± SD	Mean ± SD	Mean ± SD	Mean ± SD
When you go to the consultation, are you treated by the same professional?	4.98 ± 0.20	4.83 ± 0.48	4.73 ± 0.62	4.58 ± 0.83	4.94 ± 0.30
If you have any doubt are you able to speak with the same professional that treated you?	4.95 ± 0.36	4.93 ± 0.35	4.75 ± 0.80	4.62 ± 1.02	4.64 ± 0.81
Does the professional understand your questions?	4.91 ± 0.47	4.88 ± 0.52	4.81 ± 0.68	4.84 ± 0.51	4.74 ± 0.72
Does the professional talk with you about other health problems?	4.09 ± 1.50	4.43 ± 1.28	2.71 ± 1.81	2.93 ± 1.70	3.20 ± 1.60
Does the professional allow sufficient time for you to talk about your doubts?	4.83 ± 0.70	4.87 ± 0.53	4.74 ± 0.88	4.51 ± 1.09	4.36 ± 1.17
Does the professional respond to your questions in a clear way?	4.92 ± 0.42	4.86 ± 0.59	4.85 ± 0.63	4.84 ± 0.58	4.71 ± 0.73
Does the professional note your complaints in the medical records?	5.00 ± 0.00	4.96 ± 0.38	4.74 ± 0.81	4.72 ± 0.93	4.91 ± 0.53
Does the professional explain about the medications used in the treatment of TB?	4.76 ± 0.83	4.90 ± 0.51	4.77 ± 0.80	4.86 ± 0.64	4.78 ± 0.62
Does the professional ask about all the medications that you are using?	4.41 ± 1.36	4.69 ± 1.02	3.59 ± 1.84	4.15 ± 1.51	3.59 ± 1.61
What is your opinion about the health team that treats you?	4.70 ± 0.52	4.61 ± 0.59	4.42 ± 0.62	4.45 ± 0.66	4.20 ± 0.81

**Total Indicator of Bond**	4.75 ± 0.83^a^	4.80 ± 0.70^a^	4.41 ± 1.24^b^	4.45 ± 1.15^b^	4.41 ± 1.12^b^

**Bond by region**	4.66 ± 0.96*			4.43 ± 1.14*	

RP - Ribeirão Preto; FS - Feira de Santana; CG - Campina Grande; SJRP - São José do Rio Preto; ITA - Itaboraí.

No statistical differences were found among averages followed by the same letter in columns according to the Tukey test, with significance set at 5%.

*Student t-test - p < 0.05

**Table 3:** Distribution of means and standard deviations of *range of services *indicators, Brazilian municipalities, 2007.

Indicators of Range of services	Southeastern cities			Northeastern cities	
	RP	SJRP	ITA	FS	CG
	Mean ± SD	Mean ± SD	Mean ± SD	Mean ± SD	Mean ± SD
Sputum examination pot for the diagnosis of TB	4.42 ± 1.35	4.64 ± 1.07	4.94 ± 0.37	4.51 ± 1.24	4.29 ± 1.39
Skin test	2.07 ± 1.65	2.82 ± 1.93	3.12 ± 1.95	3.09 ± 1.93	3.08 ± 1.79
Examination for HIV/AIDS	4.30 ± 1.47	4.44 ± 1.35	4.38 ± 1.39	2.87 ± 1.96	3.00 ± 1.78
Monthly sputum examination pot for TB control	3.93 ± 1.53	4.03 ± 1.56	4.76 ± 0.78	4.21 ± 1.50	3.86 ± 1.63
Monthly consultation for TB control and treatment	4.93 ± 0.41	4.88 ± 0.62	4.93 ± 0.50	4.66 ± 1.06	4.80 ± 0.76
Consumer basket or food vouchers	4.15 ± 1.62	3.89 ± 1.64	4.47 ± 1.08	2.65 ± 1.94	1.06 ± 0.30
Transport vouchers	2.51 ± 1.90	2.71 ± 1.88	1.49 ± 1.28	2.04 ± 1.67	1.73 ± 1.33
Information about TB and its treatment	4.57 ± 1.10	4.86 ± 0.62	4.51 ± 1.19	4.42 ± 1.22	3.58 ± 1.53
Health education (information about other health issues)	3.40 ± 1.86	2.67 ± 1.69	2.75 ± 1.88	1.88 ± 1.39	1.38 ± 1.02
Home visits during treatment	3.72 ± 1.47	2.94 ± 1.68	3.21 ± 1.85	2.50 ± 1.76	2.01 ± 1.66
Home visits for other reasons than TB	1.16 ± 0.63	1.16 ± 0.61	1.99 ± 1.67	2.25 ± 1.68	1.71 ± 1.46
Participation in TB patient groups at the HU	1.05 ± 0.41	1.05 ± 0.29	1.36 ± 1.11	1.12 ± 0.69	1.04 ± 0.39
Directly observed therapy	3.55 ± 1.52	3.83 ± 1.40	3.02 ± 1.45	1.08 ± 0.56	1.41 ± 1.06

**Total Indicator of Range of services**	3.37 ± 1.85^a^	3.38 ± 1.84^a^	3.46 ± 1.84^a^	2.87 ± 1.91^b^	2.53 ± 1.82^c^

**Range of services by region**	3.40 ± 1.84*			2.70 ± 1.87*	

RP - Ribeirão Preto; FS - Feira de Santana; CG - Campina Grande; SJRP - São José do Rio Preto; ITA - Itaboraí.

No statistical differences were found among averages followed by the same letter in columns according to the Tukey test, with significance set at 5%.

*Student t-test - p < 0.05

**Table 4:** Distribution of means and standard deviations of *coordination *indicators, Brazilian municipalities, 2007.

Indicators of Coordination	Southeastern cities			Northeastern cities	
	RP	SJRP	ITA	FS	CG
	Mean ± SD	Mean ± SD	Mean ± SD	Mean ± SD	Mean ± SD
Does the professional from the HU get your medical records during the consultation?	4.97 ± 0.30	4.89 ± 0.50	4.88 ± 0.61	4.97 ± 0.22	4.85 ± 0.70
When you need the results of your examinations are they available at the HU?	4.87 ± 0.69	4.73 ± 1.82	4.88 ± 0.50	4.42 ± 1.26	4.55 ± 1.00
Are you advised about scheduling your follow-up appointment at the HU?	4.96 ± 0.40	4.93 ± 0.49	4.86 ± 0.72	4.92 ± 0.56	4.84 ± 0.66

**Total Indicator of Coordination**	4.93 ± 0.49^a^	4.85 ± 0.62^ab^	4.87 ± 0.61^ab^	4.77 ± 0.84^b^	4.75 ± 0.81^b^

**Coordination by region**	4.88 ± 0.58*			4.76 ± 0.83*	

RP - Ribeirão Preto; FS - Feira de Santana; CG - Campina Grande; SJRP - São José do Rio Preto; ITA - Itaboraí.

No statistical differences were found among averages followed by the same letter in columns according to the Tukey test, with significance set at 5%.

*Student t-test - p < 0.05

**Table 5:** Distribution of means and standard deviations of *family focus *indicators, Brazilian municipalities, 2007.

Indicators of Family focus	Southeastern cities			Northeastern cities	
	RP	SJRP	ITA	FS	CG
	Mean ± SD	Mean ± SD	Mean ± SD	Mean ± SD	Mean ± SD
Does the professional ask for information about your living conditions and those of your family?	3.31 ± 1.87	4.02 ± 1.54	2.30 ± 1.72	2.76 ± 1.75	2.74 ± 1.54
Does the professional ask for information about your family's diseases?	4.03 ± 1.62	4.17 ± 1.36	2.80 ± 1.83	3.24 ± 1.75	3.45 ± 1.40
Does the professional ask if the people you live with have coughs, fever?	4.39 ± 1.36	4.23 ± 1.38	4.01 ± 1.58	3.88 ± 1.62	3.80 ± 1.42
When you became ill with TB did the professional deliver sputum examination pots to everyone that lives with you?	2.91 ± 1.98	3.81 ± 1.67	3.69 ± 1.89	2.89 ± 1.92	2.22 ± 1.55
Does the professional know the people that live with you?	4.04 ± 1.61	3.64 ± 1.60	4.11 ± 1.67	3.09 ± 1.68	2.76 ± 1.72
Do the professionals speak to the people that live with you about your disease?	3.75 ± 1.73	3.48 ± 1.65	3.09 ± 1.91	2.82 ± 1.70	2.54 ± 1.63
Do the professionals speak to the people that live with you about your treatment?	3.72 ± 1.80	3.50 ± 1.67	3.05 ± 1.87	2.84 ± 1.77	2.46 ± 1.59
Do the professionals speak to the people that live with you about other health problems?	3.05 ± 1.90	2.11 ± 1.55	2.00 ± 1.65	1.73 ± 1.41	1.92 ± 1.46

**Total Indicator of Family focus**	3.65 ± 1.80^a^	3.62 ± 1.68^a^	3.13 ± 1.90^b^	2.91 ± 1.79^bc^	2.74 ± 1.64^c^

**Family focus by region**	3.47 ± 1.81*			2.82 ± 1.72*	

RP - Ribeirão Preto; FS - Feira de Santana; CG - Campina Grande; SJRP - São José do Rio Preto; ITA - Itaboraí.

No statistical differences were found among averages followed by the same letter in columns according to the Tukey test, with significance set at 5%.

*Student t-test - p < 0.05

The dimension "access to treatment" was evaluated as satisfactory in the three Southeastern cities (RP, SJRP and ITA) and regular in the two Northeastern municipalities (FS and CG). 'Need for motorized transportation' was unsatisfactory in all cities. 'Expense with transportation' was also a problem in ITA and FS. Northeastern cities (FS and CG) presented poor results in 'domiciliary visits' and 'distance between treatment site and patient's house' (table 1).

The dimension "bond" was assessed as satisfactory in all cities, with a slightly better performance in RP and SJRP. The indicator 'professionals talk about other health problems' was evaluated as regular in FS, CG and ITA (table 2).

The dimension "range of services" was rated as regular, with better performance of Southeastern cities (RP, SJRP and ITA). The offering of 'home visits for other reasons than TB' and 'patients' support groups' showed an unsatisfactory evaluation in all study sites. In RP, the 'tuberculosis skin test' was also rated as unsatisfactory. ITA, FS and CG have shown problems with 'transport vouchers'. 'Health education', 'DOT' and 'food vouchers' were less offered in the Northeastern cities (table 3).

The dimension "coordination' was evaluated as satisfactory in all cities (table 4).

The dimension "family focus" was evaluated as satisfactory in RP and SJRP, and regular in ITA, FS and CG. 'Professional asking patient's family about other health problems' was evaluated as unsatisfactory, except in RP. In ITA, professionals almost never 'ask patients about their life conditions'. In CG, the 'delivery of sputum pot at patients' home' and 'orientation about TB by professionals to patients' family' were also evaluated as unsatisfactory (table 5).

## Discussion

The results regarding the socio-demographic profile of the TB patients interviewed seem to be similar to those found in most national and international scientific literature, reinforcing the relationship between the disease and social vulnerability [16], demonstrating that the disease mostly affects males and individuals with intermediate education levels [17-19].

The organization of TB patient care in the study cities presented heterogeneity, with treatment coexisting at two types of health services, predominantly medical consultations for treatment at TRC. Some authors reveal that regional disparities and social inequality are strong elements that corroborate the diversity in care delivery in the Brazilian scenario [4,20].

The expansion of the FHS by itself does not guarantee the sustainability of TB control actions in PHC, but a political commitment of managers is necessary [21], through an integrated approach in the health system, which requires permanent and sustained PHC actions [22]. High coverage of FHS did not influence cities' performance in TB care.

The frequency and site where DOT is performed varied among cities, with greater DOT coverage in cities in the Southeast. Of the five cities, only one (RP) provided DOT at the patients' home. The other cities with higher FHS coverage could present a better performance; however, treatment is conducted at TRC, without establishing partnerships with PHC services, which are provided with Community Health Workers to accomplish this activity. Studies in Brazil have identified that there is a shortage of financial and human resources with appropriate profile to work focused on a community approach and operational difficulties in the use of DOT for most patients under treatment [23].

In this study, predominance of TB treatment at TRC was determinant for poor performance on some "access to treatment" indicators. Such indicators could be addressed through the insertion of TB program actions at PHC services, which are closest to patients' home. Although the medication is free of charge, indirect costs and losses, such as transport and wages lost, respectively, may turn treatment unfeasible and follow-up difficult [24].

To overcome this impasse, some countries propose a health system that combines an outpatient referral unit, a defined team for specialized management at district level, which is responsible for treatment, supervision and monitoring, including PHC participation and responsibility in case search and Directly Observed Therapy (DOT) supervision activities. They also recommend a balance between integration, specificity and decentralization and centralization functions, as well as the inclusion of innovative approaches for specialized groups [25].

All study sites achieved a satisfactory performance on indicators for the dimension "bond". As TRC professionals attend to specific clients in response to a programmed demand, they can dedicate more time to individual case management and understanding of TB patients' singularity. These aspects permit the development of shared responsibilities between patients and health care professionals and the acknowledgement of individual subjectivities involved in the care process [26] and indicate that the "bond" could be more related to the relationship the user establishes with the health service than to the service location in the geographic area.

The dimension "range of services" was evaluated as regular in the five cities, showing that patients face a lack of social support and collective actions. In daily TB treatment situations, even simple cases require the involvement of great care complexity. They demand epidemiological surveillance, clinical actions supported by therapeutic techniques and by integration between individual and collective care, curative and preventive actions, and care and educative activities [27]. Health teams' performance requires more than a clinical approach, and also needs a policy that guarantees the insertion of TB control actions in the health system [28].

The dimension "coordination" was well evaluated in its three indicators and reflects health care professionals' concern related to patient follow-up. For a more detailed analysis of this dimension, other elements are required, such as the analysis of health care service integration, information system quality and reference mechanisms.

The accomplishment of family-focused actions was evaluated as regular in the cities with high PHC coverage, in contrast with a better evaluation in RP and SJRP. This fact highlights the need to involve family members in the TB patient care process and also include them as an object of care. PHC professionals can accomplish this, have this responsibility and should be included in TB care.

Despite facing the significant implantation and expansion process of the FHS in Brazil, the dominance of the traditional healthcare model is still clear [29,30]. In the country, tools to encourage the incorporation of epidemiological surveillance actions in the PHC context "are not always directed to induce a greater integration of the various levels of complexity of care" [31].

The transition from the conventional model of the TRC to the PHC services was identified in some cities, as well as the importance of integrating both. The decentralization of TB control actions to PHC in countries whose health systems have not been consolidated yet demands caution, as it can result in diluted responsibilities, lack of commitment, low quality or lack of laboratory support, fragmentation of treatment regimens and programs, difficulties to accomplish DOT and flaws in information systems capable of providing reliable reports [32].

Therefore, TRC should act as a support for training, supervision, monitoring and evaluating TB care at the different health system levels. Thus, specific technologies for TB patient treatment and care could be guaranteed, integrating them with other PHC attributes and promoting co-responsibility at all healthcare levels. The struggle for political spaces, ideological and technological aspects [33] remain as obstacles for the achievement of health service integration.

Some limitations and difficulties that were identified in this study are related to the organizational characteristics of the health system and TB care in the cities and to the method (sample size for each city, interviews in violent areas that required the presence of a health care professional, difference in treatment conduction among sites (centralized and decentralized services) and the instrument that needed to be readapted for each treatment organization mode. An operational and epidemiological TB research group enhanced the project development, which works in an integrated way within cities and health services.

## Conclusions

One can affirm that two types of obstacles exist for health service performance in TB treatment in the cities under analysis, mainly in the Northeast. The first is structural and derives from difficulties to access health services and actions. The second is organizational and derives from the way health technologies and services are distributed and integrated. Incentives to improve care organization and management practices, including the integration of primary, secondary and tertiary services, can contribute towards a better performance of health services, and mainly PHC, in TB treatment.

## Competing interests

The authors declare that they have no competing interests.

## Authors' contributions

TCSV coordinated the writing of the manuscript, was the principal investigator, had the concept and design of the study and performed data analysis and discussion; ARN had the concept and design of the study and performed data analysis and discussion; LMS performed data analysis and discussion; RLPA drafted the paper, participated in data collection and performed data analysis and discussion; MEFB and TA participated in data collection and performed data analysis and discussion; JAN, PFP, LDS, MMAS and SHFV participated in data collection and performed data analysis and discussion; AAM and RAA participated in data collection and performed data analysis and discussion. All authors contributed to the interpretation of results, read and approved the final manuscript.

## Pre-publication history

The pre-publication history for this paper can be accessed here:

http://www.biomedcentral.com/1472-6963/11/241/prepub
